# Gut Check: Do Interactions between Environmental Chemicals and Intestinal Microbiota Affect Obesity and Diabetes?

**DOI:** 10.1289/ehp.120-a123a

**Published:** 2012-03-01

**Authors:** Wendee Holtcamp

**Affiliations:** Wendee Holtcamp is a science writer based in Houston, Texas.

The microbiota that populate human intestinal tracts vary substantially from person to person, and mounting evidence suggests these interindividual variations in gut microbiota affect how a person metabolizes chemicals they may be exposed to. A review of the literature on this topic directed attention to a new hypothesis: that interactions between gut ecology and environmental chemicals contribute to obesity and diabetes [*EHP* 120(3):332–339; Snedeker and Hay]. No study has yet directly addressed that hypothesis, but this review comments on the strengths and weaknesses of studies linking environmental chemicals to obesity and diabetes and identifies gaps in the knowledge of how gut microbiota may affect the metabolism of these chemicals.

Studies reviewed by the authors found that differences in gut microbiota affected the toxicity of certain pharmaceuticals, including acetaminophen and the chemotherapy medication CPT-11. The authors propose that the same mechanisms may be at work with environmental chemicals. Enzymes produced by different gut microbe species can render ingested chemicals either more or less bioavailable, thereby affecting their toxicity. In reviewing evidence on 3 dozen suspected obesogenic and diabetogenic chemicals, the authors found that at least three-quarters of them may be metabolized by gut microbe enzymes in a way that affects their absorption, distribution, metabolism, and excretion.

**Figure:**
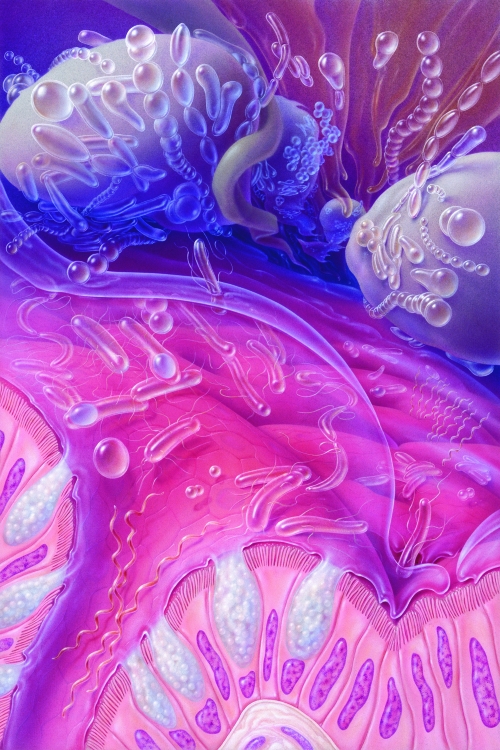
An individual’s unique gut microbiota profile may affect how that person metabolizes environmental chemicals. © Jane Hurd/National Geographic Stock

Human studies have shown that obese and diabetic individuals have different gut microbiota compositions than lean and nondiabetic individuals. Likewise, bariatric surgery was associated with altered gut microbiota. The composition of a gut’s microbiome may influence body weight by regulating fat storage, altering the ability of the intestines to extract energy from food, and affecting satiety by modulating the levels of hormones that regulate appetite.

The reviewers also found developmental links between gut microbiota and obesity. For instance, one study connected microbiome composition of children to their mother’s weight, body mass index, and degree of weight gain during pregnancy, while another linked differences in infant microbiota composition to weight gain years later. The review highlights the importance of understanding how differences in gut microbiota might affect the fate of environmental chemicals in people and influence human health outcomes.

